# Epigenetic signatures of Werner syndrome occur early in life and are distinct from normal epigenetic aging processes

**DOI:** 10.1111/acel.12995

**Published:** 2019-07-01

**Authors:** Anna Maierhofer, Julia Flunkert, Junko Oshima, George M. Martin, Martin Poot, Indrajit Nanda, Marcus Dittrich, Tobias Müller, Thomas Haaf

**Affiliations:** ^1^ Institute of Human Genetics Julius Maximilians University Würzburg Germany; ^2^ Department of Pathology University of Washington Seattle Washington USA; ^3^ Department of Clinical Cell Biology and Medicine, Graduate School of Medicine Chiba University Chiba Japan; ^4^ Department of Bioinformatics Julius Maximilians University Würzburg Germany

**Keywords:** (classical and atypical) Werner syndrome, bisulfite pyrosequencing, methylation array, premature aging, segmental progeria, transcription deficiency

## Abstract

Werner Syndrome (WS) is an adult‐onset segmental progeroid syndrome. Bisulfite pyrosequencing of repetitive DNA families revealed comparable blood DNA methylation levels between classical (18 *WRN*‐mutant) or atypical WS (3 *LMNA*‐mutant and 3 *POLD1*‐mutant) patients and age‐ and sex‐matched controls. WS was not associated with either age‐related accelerated global losses of ALU, LINE1, and α‐satellite DNA methylations or gains of rDNA methylation. Single CpG methylation was analyzed with Infinium MethylationEPIC arrays. In a correspondence analysis, atypical WS samples clustered together with the controls and were clearly separated from classical WS, consistent with distinct epigenetic pathologies. In classical WS, we identified 659 differentially methylated regions (DMRs) comprising 3,656 CpG sites and 613 RefSeq genes. The top DMR was located in the *HOXA4* promoter. Additional DMR genes included *LMNA*, *POLD1*, and 132 genes which have been reported to be differentially expressed in *WRN*‐mutant/depleted cells. DMRs were enriched in genes with molecular functions linked to transcription factor activity and sequence‐specific DNA binding to promoters transcribed by RNA polymerase II. We propose that transcriptional misregulation of downstream genes by the absence of WRN protein contributes to the variable premature aging phenotypes of WS. There were no CpG sites showing significant differences in DNA methylation changes with age between WS patients and controls. Genes with both WS‐ and age‐related methylation changes exhibited a constant offset of methylation between *WRN*‐mutant patients and controls across the entire analyzed age range. WS‐specific epigenetic signatures occur early in life and do not simply reflect an acceleration of normal epigenetic aging processes.

## 1 INTRODUCTION

Aging is a universal biological process, leading to an overall decline of organ functions, tissue homeostasis, and the ability to successfully respond to internal and external stresses, which takes place at highly different rates within members of a species and between species. Segmental progeroid syndromes are very rare monogenic human disorders showing clinical features of premature aging involving more than one tissue or organ (Martin, 1978). Werner syndrome (WS; OMIM 277700) is an autosomal recessive adult‐onset segmental progeria that is characterized by ocular cataracts, scleroderma‐like skin changes, subcutaneous calcification and ulceration, premature graying and loss of hair, short stature from the second decade of life, and an elevated risk for age‐associated diseases such as atherosclerosis, diabetes mellitus, and osteoporosis. Cancer (especially sarcomas) and myocardial infarction are the leading causes for early death at an average age of 54 years.

More than 80 different homozygous or compound heterozygous mutations in the Werner syndrome (*WRN)* gene have been associated with WS (Yokote et al., 2017). The WRN protein is a member of the RecQ family of helicases possessing both 3'‐>5' DNA helicase and 3'‐>5' exonuclease activities (Yu et al., 1996). Cells from WS patients show a prolonged S phase of the cell cycle, hypersensitivity to agents causing DNA crosslinks and double‐strand breaks, elevated frequencies of micronuclei, and a reduction in recombinational double‐strand break repair (Dhillon et al., 2007; Poot, Jin, Hill, Gollahon, & Rabinovitch, 2004). WS fibroblasts show a limited proliferative lifespan due to clonal attenuations and successions, a variegated chromosomal translocation mosaicism, and multiple spontaneous deletions (Fukuchi, Martin, & Monnat, 1989; Salk, Au, Hoehn, & Martin, 1981). It has therefore been hypothesized that *WRN* plays a role in the resolution of potentially damaging, complex DNA structures accidentally formed during DNA replication, recombination, repair, and transcription as well as in preventing chromothripsis (Poot, 2018).

Atypical WS is characterized by a WS‐like phenotype without *WRN* mutations. Some patients with atypical WS carry heterozygous mutations in the lamin A (*LMNA*) gene (Chen et al., 2003). These nuclear intermediate filaments are major structural components of the mammalian nuclear lamina, contributing to nuclear shape, mechanical stability, nuclear assembly, and positioning. They are involved in chromatin organization, transcription regulation, and DNA replication (Mattout, Dechat, Adam, Goldman, & Gruenbaum, 2006). Ten to 15% of patients initially diagnosed with WS displayed mutations neither in *WRN* nor in *LMNA*. A subset of them presented with mandibular hypoplasia, deafness, and progeroid features (MDPL syndrome) and heterozygous mutations in the polymerase delta 1 (*POLD1*) gene (Weedon et al., 2013). POLD1 has both DNA polymerase and 3'‐>5' exonuclease activities and is involved in DNA synthesis of the lagging strand, mismatch repair, and resolution of DNA replication‐blocking structures. It functionally and physically interacts with WRN during DNA replication and repair (Kamath‐Loeb, Shen, Schmitt, & Loeb, 2012). Skin fibroblasts of a MDPL patient exhibited increased fractions of senescent markers and persistent DNA damage after genotoxic treatment (Fiorillo et al., 2018).

Although in most segmental progerias the underlying mutations are known, the molecular mechanisms causing a plethora of aging‐like phenotypes remain to be elucidated. Impairment of genome stability explains some symptoms; however, other mechanisms, in particular epigenetic dysregulation, may also play important roles, as WS fibroblasts and WRN‐depleted cells show extensive alterations of gene expression (Cheung et al., 2014; Kyng, May, Kolvraa, & Bohr, 2003; Zhang et al., 2015). The most thoroughly studied epigenetic modification is DNA methylation at the carbon 5’ atom of cytosine, mainly in the context of CpG dinucleotides. Methylation of CpG islands, which are present in the promoter and/or first exon of most mammalian genes, leads to an inactive chromatin structure and gene silencing during development, differentiation, and disease. In contrast, gene body methylation is usually associated with active genes (Jones, 2012). Methylated CpGs are enriched in repetitive DNA elements to prevent retrotransposition and to maintain genome integrity (Yoder, Walsh, & Bestor, 1997). There is to date only limited information on DNA methylation patterns associated with premature aging diseases. One methylation array study (Heyn, Moran, & Esteller, 2013) compared lymphoblasts of four patients with WS (two with *WRN*, one with *LMNA*, and one without a known mutation) and three related nonmutant patients with Hutchinson–Gilford progeria with normal lymphoblasts, naive B cells, and peripheral blood mononuclear cells. The samples with *WRN* and *LMNA* mutations clustered together and were distinct from nonmutant patients and controls. A conceptually related study (Guastafierro et al., 2017) found profound blood methylation differences between three classical WS patients and controls; however, these results were not statistically significant. We now report a more comprehensive methylome analysis of 24 independent patients with segmental progeria (18 with *WRN*, 3 with *LMNA*, and three with *POLD1* mutations) together with carefully matched controls.

## 2 RESULTS

### 2.1 Global DNA methylation of repetitive elements in WS

Aging is associated with hypo‐ and hypermethylation events at specific regions of the genome (Unnikrishnan et al., 2018). Recently, we showed that the methylation of various repeat families decreased, whereas that of rDNA increased during in vitro aging of fibroblast clones (Flunkert et al., 2018). Here, we used the same bisulfite pyrosequencing assays to quantify mean methylation of ALU, LINE1, and α‐satellite repeats in *WRN*‐, *LMNA*‐, and *POLD1*‐mutant patients and controls (Table 1). Both interspersed repeats and centromeric α‐satellite DNA showed almost identical methylation levels in WS patients and controls. Methylations of the rDNA promoter and upstream control element were increased by 1–2 percentage points in *WRN*‐ and *POLD1*‐mutant patients and decreased by approximately 5% in *LMNA*‐mutant patients (Table 1); however, due to the large interindividual variations these results were not significant (Table S1).

**Table 1:** Mean methylation of repetitive elements in whole blood samples of WS patients and matched controls

Mutant gene	Disease status	α‐satellite DNA			ALU repeats			LINE1 repeats			rDNA promoter			rDNA prom./UCE		
		*N*	Mean	*SE*	*N*	Mean	*SE*	*N*	Mean	*SE*	*N*	Mean	*SE*	*N*	Mean	*SE*
*WRN*	Controls	18	88.4	0.4	18	25.0	0.3	17	83.9	0.3	17	17.6	1.0	18	25.4	1.3
	Patients	18	88.4	0.3	18	25.3	0.1	18	84.2	0.3	18	19.8	1.4	18	27.9	1.9
*LMNA*	Controls	3	88.7	0.6	3	25.6	0.3	3	83.5	1.0	3	22.2	3.2	3	30.9	3.7
	Patients	3	88.6	0.6	3	25.4	0.2	3	83.2	0.7	3	18.0	1.0	3	24.7	1.7
*POLD1*	Controls	3	89.3	0.6	3	24.8	1.1	3	83.4	0.6	2	17.1	1.8	3	28.7	0.7
	Patients	3	89.5	0.4	3	25.2	0.8	3	84.1	0.8	2	18.6	0.6	3	28.8	3.5

### 2.2 Differentially methylated sites and regions in WS

**Figure 1:**
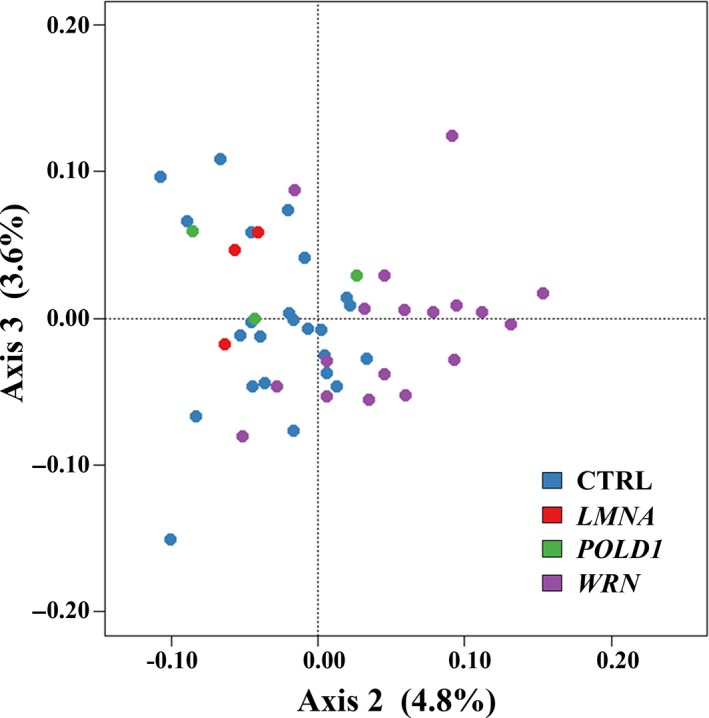
Correspondence analysis of the 10,000 most variable CpG sites over all 48 blood samples. Clear separation of classical WS from the remaining samples on the second axis explains 4.8% of the variance. The samples from *LMNA*‐ and *POLD1*‐mutant patients cluster with the controls

**Figure 2:**
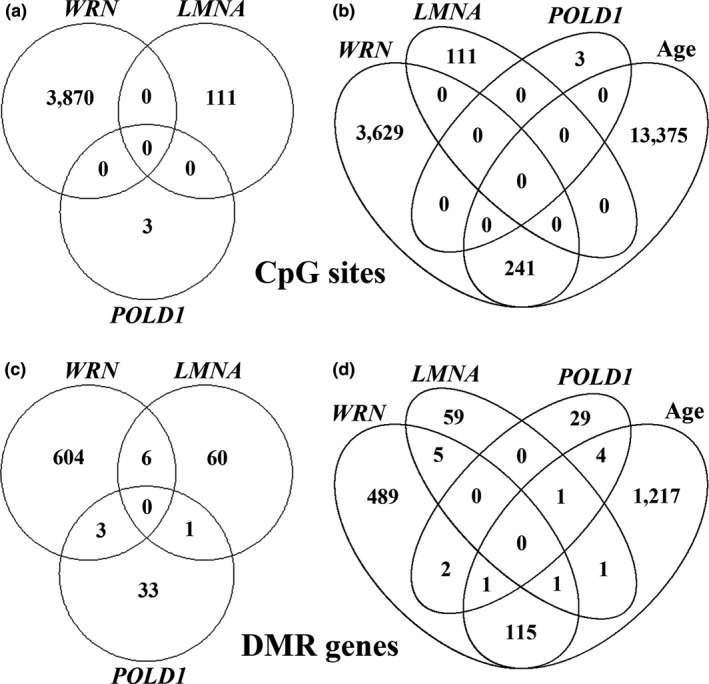
Venn diagrams showing the overlaps of genome‐wide significant CpG sites (a, b) and DMR‐containing genes (c, d) between *WRN*‐, *LMNA*‐, and *POLD1*‐mutant patients (a, c) as well as between WS‐ and age‐related changes (b, d)

Infinium MethylationEPIC BeadChips were used to compare genome‐wide DNA methylation patterns at a single CpG level between WS patients and controls. After initial filtering, 816,980 probes were included in the analysis. Although estimations of the proportions of blood cell types did not reveal significant differences between patient and control samples (Figure S1), exploratory analyses clearly indicated cell composition as a major factor explaining array methylation variation (Figure S2). Therefore, these scores were included into the linear model. A correspondence analysis of the 10,000 most variable methylation sites over all 48 samples clearly separated (axis 2; 4.8%) the *WRN*‐mutant patients from the remaining samples (Figure 1). The 3 *LMNA*‐mutant and the 3 *POLD1*‐mutant atypical WS patients were located inside the control cluster. At the single CpG level, 3,870 of 812,996 analyzed array CpGs showed a significant (adjusted *p* < 0.05) methylation difference in *WRN*‐mutant, 111 in *LMNA*‐mutant, and three in *POLD1*‐mutant patients, compared with controls. There was not a single CpG site with genome‐wide significance overlapping between patient groups (Figure 2a).

**Figure 3:**
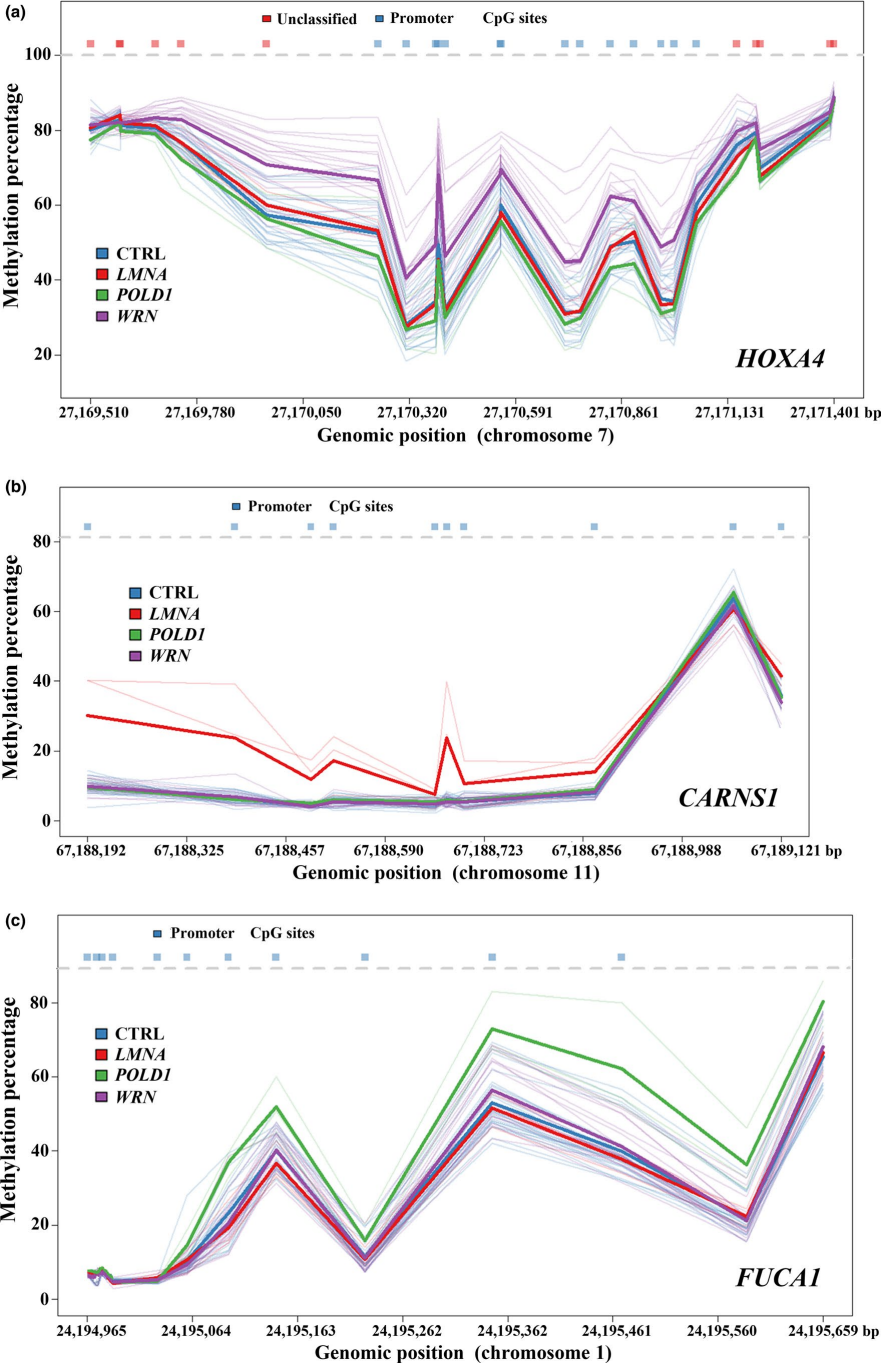
Top promoter DMRs in *WRN*‐mutant (a), *LMNA*‐mutant (b), and *POLD1*‐mutant (c) patients. Thick colored lines represent the methylation profile of the analyzed DMR in controls (blue), *WRN*‐mutant (mauve), *LMNA*‐mutant (red), and *POLD1*‐mutant (green) patients. The genomic region is indicated on the x‐axis and individual CpG sites within the DMR on the top. The y‐axis indicates the methylation level at a given genomic position. Thin colored lines represent methylation profiles of individual samples, while the solid line indicates the mean methylation level per group. *HOXA4* (a) is hypermethylated in classical WS patients; *LMNA*‐ and *POLD1*‐mutant patients behave similar to controls. The *CARNS1* promoter (b) is hypermethylated in *LMNA*‐mutant patients and the *FUCA1* promoter (c) in *POLD1*‐mutant patients

In total, 659 differentially methylated regions (DMRs) encompassing 3,656 CpGs and 613 RefSeq genes exhibited genome‐wide significant methylation differences between 18 *WRN*‐mutant patients and controls (Table S2a). The majority (78%) of these blood DMRs were hypermethylated in WS patients. The top 25 DMRs are listed in Table 2. As a graphical example, Figure 3a presents the methylation profile of the *HOXA4* promoter region, which was hypermethylated in classical but not in atypical WS, compared with controls. Enrichment analysis identified six significant Gene Ontology (GO) terms (Table 3), including one biological process for intercellular signal transduction and three molecular functions related to transcription factor activity and sequence‐specific DNA binding. Analysis of a much smaller published EPIC array data set (GSE100825) using our bioinformatics pipeline did not yield significantly differentially methylated CpGs. However, the *β* differences between WS patients and controls correlated well between the published study (Guastafierro et al., 2017) and our study (*r* = 0.182, *p* < 0.001), consistent with a common signal in both data sets. From previous studies (Table S3), we obtained six partially overlapping lists of genes that were differentially expressed between WS patient‐derived or *WRN*‐depleted cells (mainly fibroblasts) and controls. 132 of our 613 DMR genes showed differential expression in at least one, 36 in at least two, and seven in at least three studies. Expression of *SGK1* was consistently upregulated, whereas *DEPDC1*, *E2F8*, *HIST1H1A*, *POLD1*, *SMC4*, and *PKMYT1* were transcriptionally downregulated in classical WS.

**Table 2:** Top 25 DMRs in classical (*WRN*‐mutant) WS patients

DMR ID	*p* Value	Chromosomal position^a^	DMR length	No. of CpGs	Mean ß value	Mean ß difference^b^	Largest ß difference^b^	Associated gene	Gene region	Relation to CpG island
1	1.01E−27	Chr7:27,169,510–27,171,401	1,891	25	0.62	0.08 ↑	0.18 ↑	*HOXA4*	Body, 1stExon, 5'UTR, TSS200, TSS1500	N_Shore, Island, S_Shore
2	1.98E−25	Chr11:312,518–316,456	3,938	26	0.44	−0.02 ↓	−0.16 ↓	*IFITM1*	TSS1500, 1stExon, 5'UTR, Body, 3'UTR	S_Shore, N_Shelf, N_Shore, Island
3	8.72E−21	Chr3:160,119,314–160,121,275	1,961	6	0.33	−0.11 ↓	−0.19 ↓	*SMC4*; *MIR15B*; *MIR16−2*	Body, Exon Bnd; TSS1500; TSS1500	S_Shore, S_Shelf
4	1.03E−20	Chr19:52,390,810–52,391,789	9,79	14	0.38	0.06 ↑	0.14 ↑	*ZNF577*	Body, 5'UTR, 1stExon, TSS200, TSS1500	N_Shore, Island, S_Shore
5	1.62E−19	Chr6:30,852,963–30,854,551	1,588	17	0.58	0.06 ↑	0.15 ↑	*DDR1*	5'UTR	S_Shore
6	5.23E−19	Chr10:104,196,206–104,196,541	335	5	0.49	−0.08 ↓	−0.11 ↓	*MIR146B*	TSS200, Body	S_Shelf, OpenSea
7	2.17E−18	Chr1:2,983,926–2,984,525	599	7	0.30	0.08 ↑	0.27 ↑	*FLJ42875*; *PRDM16*	Body, TSS200, TSS1500; TSS1500	Island
8	6.90E−18	Chr18:11,146,255–11,147,785	1,530	4	0.50	0.08 ↑	0.11 ↑	*FAM38B*	Body	N_Shelf, N_Shore
9	5.22E−17	Chr11:32,451,777–32,455,025	3,248	20	0.38	0.04 ↑	0.08 ↑	*WT1*	Body, TSS200, TSS1500	N_Shore, Island, S_Shore
10	4.15E−15	Chr4:174,202,697–174,203,520	823	6	0.55	−0.08 ↓	−0.10 ↓	*GALNT7*	Body	OpenSea
11	9.80E−15	Chr6:31,846,769–31,847,028	259	8	0.59	0.07 ↑	0.11 ↑	*SLC44A4*	5'UTR, 1stExon, TSS200, TSS1500	OpenSea
12	1.33E−14	Chr2:65,593,761–65,594,021	260	5	0.35	−0.09 ↓	−0.16 ↓	*SPRED2*	5'UTR, 1stExon, Body, TSS200	OpenSea
13	5.23E−14	Chr14:104,170,490–104,172,224	1,734	9	0.57	0.05 ↑	0.08 ↑	*XRCC3*	Body	OpenSea
14	1.71E−13	Chr17:935,017–935,235	218	4	0.62	0.10 ↑	0.13 ↑	*ABR*	Body, TSS200, TSS1500	Island, S_Shore
15	2.14E−13	Chr9:100,069,294–100,070,142	848	10	0.61	0.06 ↑	0.11 ↑	*CCDC180; KIAA1529*	TSS1500, TSS200; Body	N_Shore, Island
16	2.95E−13	Chr5:163,723,456–163,724,070	614	10	0.36	0.06 ↑	0.10 ↑			OpenSea
17	3.24E−13	Chr3:22,412,124–22,412,963	839	4	0.61	0.13 ↑	0.18 ↑			N_Shore
18	4.06E−13	Chr6:30,652,907–30,653,799	892	12	0.26	−0.04 ↓	−0.09 ↓	*PPP1R18*	Body, 1stExon, 5'UTR	N_Shore
19	5.89E−13	Chr3:156,838,096–156,838,403	307	5	0.64	0.07 ↑	0.11 ↑			Island, S_Shore
20	1.09E−12	Chr6:29,454,623–29,454,954	331	6	0.50	0.05 ↑	0.10 ↑	*MAS1L*	1stExon	OpenSea
21	1.17E−12	Chr8:8,746,679–8,747,452	773	4	0.55	−0.07 ↓	−0.11 ↓	*MFHAS1*	Body	N_Shore
22	1.85E−12	Chr2:204,801,413–204,801,510	97	4	0.33	−0.09 ↓	−0.11 ↓	*ICOS*	TSS200, 5'UTR, 1stExon	OpenSea
23	2.46E−12	Chr1:1,564,422–1,565,931	1,509	10	0.28	0.05 ↑	0.11 ↑	*MIB2*	Body, 3'UTR	Island
24	3.38E−12	Chr13:48,986,124–48,987,465	1,341	4	0.58	−0.08 ↓	−0.10 ↓	*LPAR6; RB1*	Body, 1stExon, 5'UTR; Body	OpenSea
25	3.39E−12	Chr4:81,118,188–81,119,473	1,285	12	0.33	0.08 ↑	0.11 ↑	*PRDM8*	TSS1500, 5'UTR, TSS200, 1stExon	Island, N_Shore, S_Shore

a Genomic coordinates are based on Ensembl release 75.

b ↑ hypermethylated; ↓ hypomethylated in WS compared with controls.

**Table 3:** Enrichment of GO terms for biological processes and molecular function in genes with DMRs

Category: Term	No. of genes	Fold enrichment	Adjusted *p* value	Genes with DMRs^a^
Biological process: intracellular signal transduction (GO 0035556)	30	2.8	0.002	*GPR182, PRKCZ, CARHSP1, NRBP1, ADCY7, TOLLIP, MAP4K2, MCF2L,* ***DGKA*** *, TIAM2,* ***NSMCE1*** *, ZAP70, SH2B2, NRG2, AKT3, S100A1, AKT2,* ***SGK1*** *, ABR,* ***SOCS2*** *,* ***DEPDC1*** *,* ***CISH*** *, TNS3, MAST4,* ***KSR2*** *,* ***RPS6KA2*** *, FYN, CHN2, TSSK6,* ***KALRN***
Molecular function: transcription factor activity, sequence‐specific DNA binding (GO 0003700)	48	1.9	0.013	*RAI1, ZNF274, E2F8, FOXK2, PAX6, RORC, NFIX, CBFA2T3, ZIC1,* ***TCF7L2*** *, WT1, ZKSCAN4, FOS, ZGPAT,* ***PRDM15*** *, HOXA4, ZNF300, PAX8, ETV2, ZNF540,* ***ARNTL2*** *, HSF4, SIM1,* ***RUNX3*** *, FOXD4, TFDP1, ZNF577, MAFF, RXRB, GTF2H4, ESR1, TEAD2,* ***RB1*** *, GRHL2,* ***HMGA1*** *, GAS7, TP73, STAT3, MYCN,* ***HOXB4*** *, FOXD4L1, HEYL, BNC1, ZNF418, MGA, IRF2, PBX1, PBX2*
Molecular function: transcriptional activator activity, RNA polymerase II core promoter proximal region sequence‐specific binding (GO 0001077)	19	3.1	0.014	*CEBPE, FOXK2, ESR1, PAX6,* ***NR4A1*** *, ZIC1, MEIS1, STAT3, WT1, TP73, FOS, EBF4,* ***BCL11B*** *, PAX8, HEYL, ETV2, TXK, PBX1,* ***SOX18***
Molecular function: protein kinase activity (GO 0004672)	24	2.6	0.014	*SGK494,* ***OBSCN*** *, PRKCZ, NRBP1, LTK, GTF2H4,* ***MAPKAPK3*** *, MAP4K2, PKMYT1, TTN, MAP3K6, DDR1,* ***KSR2*** *, HIPK1,* ***RPS6KA2*** *, HSPB8, ULK3, LMTK3, TXK, TNK2, TSSK6, AKT3, AKT2,* ***KALRN***
Molecular function: Rho guanyl‐nucleotide exchange factor activity (GO 0005089)	10	5.0	0.025	***OBSCN*** *, ABR, TIAM2, TIAM1,* ***PLEKHG5*** *, ARHGEF15,* ***ARHGEF10*** *, MCF2L, FARP2,* ***KALRN***
Molecular function: RNA polymerase II core promoter proximal region sequence‐specific DNA binding (GO 0000978)	22	2.4	0.049	*ZNF536,* ***NACC2*** *, CEBPE, E2F8, FOXK2, ESR1, PAX6, ZIC1, MEIS1,* ***TCF7L2*** *, TP73, STAT3, FOS, ZGPAT, SKOR1,* ***BCL11B*** *, PAX8, ETV2, TXK, PBX1,* ***SOX18*** *, ZNF876P*

a Genes highlighted in bold contain both hypo‐ and hypermethylated DMRs.

In *LMNA*‐mutant patients, we identified 72 genome‐wide significant DMRs encompassing 399 CpG sites and 67 RefSeq genes (Table S2b). The top promoter DMR was associated with the *CARNS1* gene (Figure 3b). Similarly, we identified 34 DMRs with 197 CpG sites overlapping with 37 RefSeq genes in *POLD1*‐mutant patients (Table S2c). The top promoter DMR was located in *FUCA1* (Figure 3c). *DKFZp761E198*, *FOXK2*, *P4HB*, *PILRB*, *STAG3L5P*‐*PVRIG2P*‐*PILRB*, and *TP73* were differentially methylated in *WRN*‐ and *LMNA*‐mutant patients; three genes, *ABR*, *ACOT7*, and *PPP1R18* (*KIAA1949*), in *WRN*‐ and *POLD1*‐mutant patients; and one gene, *EVI5L*, between *LMNA*‐ and *POLD1*‐mutant patients (Figure 2c).

### 2.3 Age‐related methylation changes

Both a site‐wise and a region‐wide analysis based on site‐wise *p* values demonstrated that age was one of the strongest contributing factors in the data set. Of 812,996 interrogated CpG sites, 13,616 (1.7%) showed genome‐wide significant age‐related methylation changes, among them 241 sites with *WRN*‐specific methylation signatures (Figure 2b). Although this is a fourfold enrichment (Fisher's exact test, *p* < 0.001), the vast majority (>90%) of differentially methylated CpGs in WS did not show age effects. There were no sites showing a significant difference in DNA methylation changes with age between WS patients and controls.

**Figure 4:**
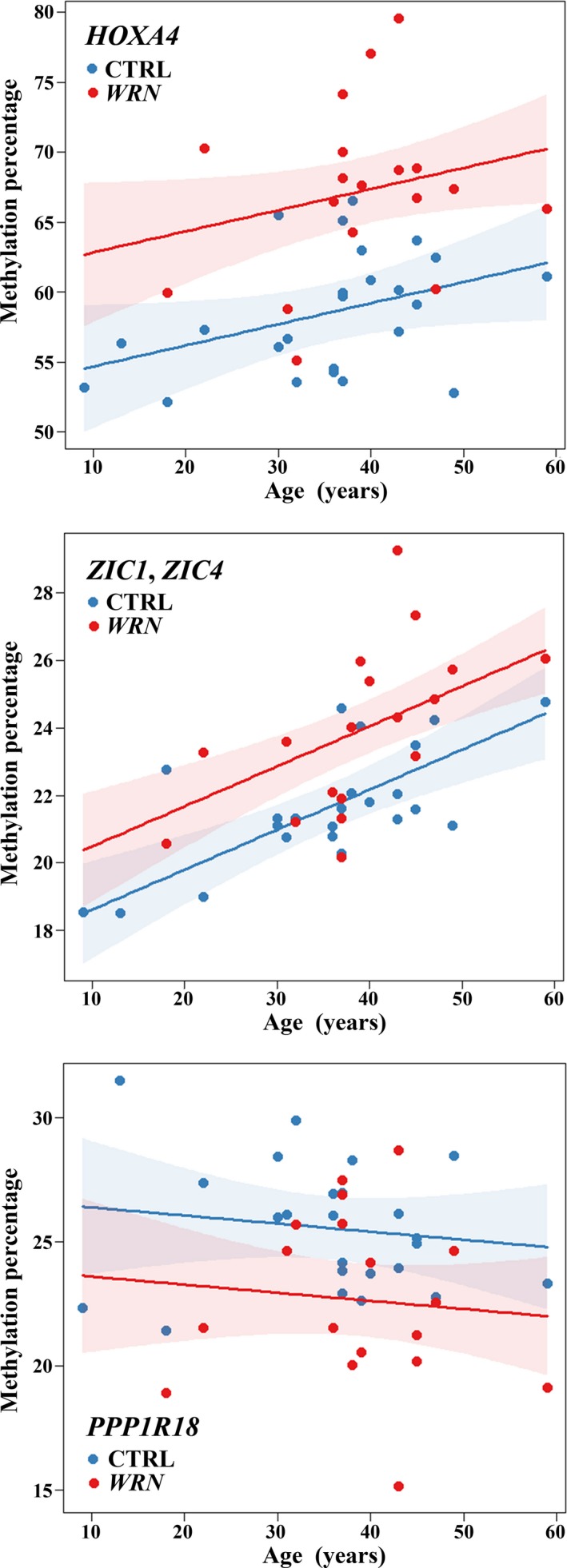
Age effect on *HOXA4*, *ZIC1*, *ZIC4*, and *PPP1R18* methylations in *WRN*‐mutant patients (red dots) and controls (blue dots). Each dot represents an individual sample; regression lines (standard errors are shaded in red and blue) indicate the methylation gains (*HOXA4*, *ZIC1*, and *ZIC4*) or losses (*PPP1R18*) of the analyzed DMR with age. Note that the methylation differences between *WRN*‐mutant patients and controls remain constant over the entire analyzed age range

Altogether, 1,340 genes were endowed with age‐related DMRs (Table S2d), 117 of which were differentially methylated in *WRN*‐mutant, three in *LMNA*‐mutant, and six in *POLD1*‐mutant patients (Figure 2d). Only two age‐dependent genes, *EVI5L* and *PPP1R18*, overlapped between *LMNA*‐ and *POLD1*‐mutant patients and between *WRN*‐ and *POLD1*‐mutant patients, respectively. The top age‐related DMR (*p* = 7.36E‐20) comprises 41 CpG sites in *ZIC1* and *ZIC4*, 16 of which define a DMR in classical WS patients. For *HOXA4*, the top DMR in *WRN*‐mutant patients, the correlation between promoter methylation and age was borderline significant (*p* = 0.0504). Most age‐dependent DMRs, including *ZIC1*, *ZIC4,* and *HOXA4,* gain methylation with age; however, there are also DMRs, for example, in the *PPP1R18* promoter losing methylation with age. When plotting DMR methylation against age (Figure 4), there was a significant additive (*ZIC1*, *ZIC4*, and *HOXA4*) or subtractive (*PPP1R18*) offset in *WRN*‐mutant patients. Across the entire age range (18–59 years), *HOXA4* was 8% (*p* = 1.01E‐27) higher, *ZIC1* and *ZIC4* 2.5% (*p* = 7.51E‐07) higher, and *PPP1R18* 4% (*p* = 4.06E‐13) lower methylated in *WRN*‐mutant patients, compared with controls. Intersections of all CpGs of age‐related DMRs with WS‐specific DMRs yielded 79 regions. Based on these regions, there were no significant interaction terms between age and methylation level after multiple testing.

## 3 DISCUSSION

### 3.1 WS is not associated with repetitive DNA methylation changes

A number of whole‐genome bisulfite sequencing (WGBS) studies have questioned the genomic hypomethylation hypothesis that gradual global demethylation of genome occurs with age (Unnikrishnan et al., 2018). Various human and mouse tissues displayed age‐related changes (both increases and decreases) in methylation at many thousands of specific CpG sites, in particular in gene‐regulatory regions; however, there was no detectable loss in global DNA methylation (Lister et al., 2013). On the other hand, most CpG sites reside in repetitive DNA elements, which may be underrepresented in WGBS data sets. The human genome contains approximately 600,000 LINE1 and more than 1,000,000 ALU retrotransposons, comprising 17% and 11% of total genomic DNA, respectively. Up to several megabases of α‐satellite DNA are present in the centromeric region of human chromosomes. Progressive loss of methylation in repetitive elements has been associated with aging and aging‐related diseases including cancer, atherosclerosis, and Alzheimer disease (Jones, Goodman, & Kobor, 2015). Hypomethylation of transposable elements leads to reactivation of retrotransposon and genome instability (Yoder et al., 1997). Although WS patients exhibit premature aging and an elevated cancer risk, we did not find evidence for hypomethylation of ALU, LINE1, and α‐satellite DNA. Bisulfite pyrosequencing is a very accurate method for the quantification of repeat methylation levels. In our experience, the variation between technical replicates (including bisulfite conversion) is of the order of one percentage point.

Ribosomal RNA constitutes the main component of the ribosome, which provides the translational machinery for protein synthesis. Several hundred rDNA transcription units are located in tandem arrays on the acrocentric short arms. However, rDNA copy number per individual varies over a range spanning at least one order of magnitude. Ribosome biogenesis is closely interrelated with cell metabolism, growth, proliferation, and the maintenance of homeostasis. Perturbations of this highly organized process cause nucleolar stress that is involved in the pathogenesis of many human diseases including cancer, and metabolic and cardiovascular disorders (Wang et al., 2015). Impaired ribosome biogenesis (Rattan, 1996) and rDNA hypermethylation (Flunkert et al., 2018) have also been linked to aging. During in vitro aging of fibroblasts, rDNA hypermethylation was reported to be more pronounced in two WS patients, compared with four controls (Machwe, Orren, & Bohr, 2000). Using the more sensitive bisulfite pyrosequencing (compared with methylation‐sensitive restriction analysis) on a larger number of blood samples, we could not confirm this result. Although *WRN*‐ and *POLD1*‐mutant patients showed slightly increased and *LMNA*‐mutant patients decreased methylation levels, there were no significant between‐group differences. Probably due to naturally occurring enormous rDNA copy number variation, the interindividual methylation variations of rDNA were considerably higher than that of the other analyzed repeat families.

### 3.2 Is classical WS a primary transcription disease?

With a prevalence of 1/50,000 (in Japan and Sardinia) to 1/200,000 (in most populations worldwide), WS is a very rare disease. Due to our increased sample sizes compared to earlier methylation array analyses (Guastafierro et al., 2017; Heyn et al., 2013), we identified genome‐wide significant DMRs in 613 RefSeq genes, the majority (78%) of which were hypermethylated in WS. The observed methylation differences were of the order of several percentage points (2%–13% for the top 25 DMRs), which is comparable to epigenetic changes associated with Down syndrome (El Hajj et al., 2016) and other genetic diseases with multiple organ dysfunction and large phenotypic variation. At the level of individual loci, there was considerable overlap in methylation variation between WS patients and controls. This is consistent with the view that multiple changes of small effect size exceeding a threshold rather than highly penetrant epimutations in a single or a few genes contribute to disease pathogenesis. A common approach to interpreting epigenetic changes in a large number of genes is gene enrichment analysis.

Genes involved in the biological process “intracellular signal transduction” were significantly enriched with DMRs. This indicates dysregulation of signal propagation to downstream components within *WRN*‐mutant cells, which then may trigger a change in cellular structure or function. Dysregulation of genes enriched in the molecular function “protein kinase activity” and “Rho guanyl‐nucleotide exchange factor activity” is known to play roles in cancer and other diseases. Most strikingly, DMRs were enriched in genes with molecular functions (three GO terms) linked to transcription factor activity and sequence‐specific DNA binding, in particular to the promoter regions of genes transcribed by RNA polymerase II (RNAPII). The assigned genes (Table 3) interact selectively and noncovalently with a specific upstream regulatory DNA sequence close to a core promoter for RNAPII, activating transcription from this promoter. It is interesting to speculate that differential DNA methylations modulating the transcriptional activities of specific subsets of genes transcribed by RNAPII contribute to the pathogenesis of WS. Dysregulation of gene expression programs that are controlled by thousands of DNA‐binding transcription factors is a hallmark of many diseases, including developmental disorders, cancer, and cardiovascular and metabolic disorders (Lee & Young, 2013). The WRN helicase appears to play a critical role in RNAPII transcription enhancement. Transcription efficiency was reduced to 40%–60% in WS cell lines (Balajee et al., 1999). Similar to Cockayne syndrome, another segmental progeria, WS is not only characterized by genome instability but also characterized by a primary RNAPII transcription deficiency. Dysregulation of gene‐regulatory networks, in particular of neuronal genes, occurs in the absence of DNA damage in Cockayne syndrome cells and may explain the severe neurological symptoms in CS patients (Wang et al., 2014).

### 3.3 Differentially methylated genes in classical WS

Our top DMR (Table 2, Figure 3a) covering 25 CpGs in the promoter‐associated region of the homeobox A4 (*HOXA4*) gene was hypermethylated (on average by 8 percentage points) in blood of WS patients. It belongs to a family of homeodomain‐containing transcription factors that play an important role during developmental processes and hematopoietic differentiation. *HOXA4* promoter hypermethylation and reduced expression have been linked to acute leukemias and a shorter survival (Strathdee et al., 2007). Hypomethylation of the *HOXA4* promoter was associated with Silver–Russell syndrome and patients with severe growth retardation of unknown etiology, suggesting a role for *HOXA4* in growth regulation (Muurinen et al., 2017). It is noteworthy that the 613 differentially methylated genes in classical WS include *LMNA* and *POLD1*, which are related to WS‐like syndromes. Other differentially methylated genes have been associated with genome instability (*RECQL5*, *TERF2*, and *XRCC3*), cataract (*EYA1*, *HSF4*, *OPA3*, *PAX6*, and *SORD*), thyroid cancer (*NDUFA13* and *PAX8*), osteoporosis (*CALCA* and *CALCR*), diabetes mellitus (*AKT2* and *TCFL2*), and diminished fertility (*ESR1*), all of which contribute to the WS aging phenotype.

Unfortunately, no RNA samples were available from our WS patients. In the literature, we found five transcriptome analyses of *WRN*‐mutant or depleted fibroblasts and of one line of mesenchymal stem cells (Table S3), but none on blood samples. It is reassuring that 132 of our 613 DMR genes in WS showed differential expression in at least one of these studies. *DEPDC1*, *E2F8*, *HIST1H1A*, *PKMYT1*, *POLD1*, and *SMC4* were transcriptionally downregulated, whereas *SGK1* was overexpressed in three or more independent studies. These genes have been linked to key cellular processes (transcription, replication, repair, cell cycle progression, chromosome condensation, stress response, apoptosis, and senescence), genome stability, and cancer; however, their possible relationship with premature aging is presently unclear.

### 3.4 WS and atypical WS are epigenetically distinct disorders

Despite small sample sizes, 67 and 37 genes with genome‐wide significant DMRs, respectively, were associated with *LMNA* and *POLD1* mutations. There was only limited overlap of differentially methylated genes between the progeroid syndromes. Six genes shared DMRs in *WRN*‐ and *LMNA*‐mutant patients, three in *WRN*‐ and *POLD1*‐mutant patients, and one in *LMNA*‐ and *POLD1*‐mutant patients. The immunoglobulin‐like type 2 receptor beta (*PILRB*) gene encodes an activating receptor which is involved in the regulation of the immune system (Wilson, Cheung, Martindale, Scherer, & Koop, 2006). *PILRB* was hypermethylated in *WRN*‐ and *LMNA*‐mutant blood, whereas in Alzheimer brain samples, it was found to be hypomethylated (Humphries et al., 2015). The active BCR‐related (ABR) protein interacts with members of the RhoGTP‐binding protein family, regulating cellular signaling (Chuang et al., 1995). *ABR* was hypermethylated in blood of *WRN*‐ and *POLD1*‐mutant patients and downregulated in WS fibroblasts (Kyng et al., 2003).

In a correspondence analysis, *LMNA*‐ and *POLD1*‐mutant patients clustered with the controls and were clearly separated from classical WS. There was not a single overlap between the 3,870 genome‐wide significant CpG sites in classical WS, 111 in *LMNA*‐mutant, and three in *POLD1*‐mutant patients. The vast majority (>98%) of differentially methylated genes in classical WS was not affected in patients with atypical WS. This may be partially due to the smaller number of atypical WS patients. Nevertheless, our data support the idea that the downstream genes whose epigenetic dysregulation may cause a premature aging phenotype largely differ between *WRN*‐, *LMNA*‐, and *POLD1*‐mutant patients. Each progeroid syndrome is associated with specific epigenetic signatures and, by extrapolation, disease pathogenesis.

### 3.5 WS and normal aging

The “epigenetic clock” is a DNA methylation‐based biomarker of aging that is defined as a weighted average across several hundred CpG sites (Horvath, 2013). The resulting epigenetic age estimate can predict lifespan and has been found to be increased in Alzheimer disease and other age‐related conditions (Horvath et al., 2018; Maierhofer et al., 2017). We previously reported an epigenetic age acceleration in blood of adult‐onset WS patients (Maierhofer et al., 2017) and in fibroblasts of childhood‐onset Hutchinson–Gilford progeria syndrome patients (Horvath et al., 2018). In contrast to the epigenetic clock, which is based on a highly selected subset (<0.05%) of array CpGs, we interrogated 816,980 CpGs representing the entire epigenome. In our data set, only 241 of 13,616 CpGs and 117 of 1,340 genes with age‐related methylation changes exhibited significant methylation differences between WS and controls. The vast majority of CpGs (3,629 of 3,870; 94%) and genes (496 of 613; 81%) with WS‐specific epigenetic signatures were not affected by aging. There was not a single CpG site showing a significant difference in methylation change with age between WS patients and controls.

Since aging is a slow and highly multifactorial process, one would not expect dramatic methylation changes (of the order of 50 or more percentage points) in individual genes in the analyzed age range. Genes with a positive (i.e., *ZIC1* and *ZIC4*) or negative correlation (i.e., *PPP1R18*) between methylation and age displayed a constant offset of methylation between *WRN*‐mutant patients and controls across the entire analyzed age range. The DMR overlapping *ZIC1* and *ZIC4* was hypermethylated (by 2.5 percentage points) in WS and gained methylation with age (0.14 percentage points per year). The transcription factors *ZIC1* and *ZIC4* are involved in brain development (Aruga & Millen, 2018) and may also have a role in bone cells. The promoter DMR in the protein phosphatase 1 regulatory subunit 18 (*PPP1R18*) was hypomethylated (by −4 and −6 percentage points, respectively) in *WRN*‐ and *POLD1*‐mutant patients and lost methylation with age (−0.11 percentage points per year). Risk SNPs in this gene have been associated with immune‐mediated diseases and blood lipid concentrations (Guo & Wu, 2019).

From an epigenetic point of view, there seems to be little in common between WS and normal aging processes. The observed methylation differences between WS and control samples most likely arose before manifestation of symptoms leading to the diagnosis of WS and remained constant during the premature aging period (from 18 to 59 years). It is interesting to speculate that during development, possibly already in the intrauterine period, WS‐specific epigenetic signatures were established. WS‐specific expression changes were reported in pluripotent stem cells (Zhang et al., 2015). Methylation changes preceding disease manifestations argue in favor of a causal relationship. We propose that epigenetic misregulation of downstream genes transcribed by RNAPII contributes to disease onset and premature aging symptoms in WS and may not be merely a secondary phenomenon. In addition to genome instability, which is a hallmark of WS, cancer, and aging, WRN may play an important role in epigenetic maintenance systems (Maierhofer et al., 2017; Zhang et al., 2015), affecting methylation patterns of hundreds of downstream genes. Collectively, our data suggest that the WS epigenome(s) is shaped early in life by processes which are largely distinct from normal epigenetic aging. Confirmations of that conclusion, however, will require comparable investigations of gene regulation during earlier stages of life.

## 4 MATERIALS AND METHODS

### 4.1 Study samples

Whole blood DNAs of 18 classical WS patients with *WRN* mutations, three atypical WS patients with *LMNA* mutations, and three MDPL patients with *POLD1* mutations (Table S4) were obtained through the International Registry of Werner Syndrome (http://www.wernersyndrome.org). Whole blood DNAs of 24 carefully age‐ and sex‐matched healthy controls were collected at the Institute of Human Genetics of Würzburg University. The EZ DNA Methylation Kit (Zymo Research) was used for sodium bisulfite conversion of 500 ng genomic DNA aliquots.

### 4.2 Bisulfite pyrosequencing

ALU, LINE1, and α‐satellite DNA repeats were first amplified in a multiplex PCR, followed by second‐round nested PCRs for each repeat. Two amplicons of the rDNA promoter were amplified separately, region 1 covering the distal rDNA promoter and region 2 the core promoter element and the upstream control element. Primer sequences and PCR conditions have been published previously (Flunkert et al., 2018). Bisulfite pyrosequencing was done on a PyroMark Q96MD pyrosequencing system (Qiagen) using the PyroMark Gold Q96 CDT reagent kit (Qiagen) and the Pyro Q‐CpG software (Qiagen). Overall methylation differences were modeled by a linear model adjusting for patient age (Table S1).

### 4.3 DNA methylation arrays

Bisulfite‐converted DNAs were whole‐genome amplified, enzymatically fragmented, and hybridized to Infinium MethylationEPIC BeadChips (Illumina). Arrays were scanned with an Illumina iScan. Raw measurements (idat files) were exported and analyzed with the statistical R framework (version 3.5.1) and the BioConductor platform (version 3.7). Preprocessing of the array data (NCBI Gene Expression Omnibus no. GSE131752) was done using the minfi package (Aryee et al., 2014). Probes overlapping known SNPs and those on the sex chromosomes were removed. In total, 816,980 probes completed all quality criteria and were used for subsequent analyses. Intensity values were normalized using the quantile normalization procedure in the minfi package. Multiple testing corrections were performed for all *p* values with the Benjamini–Hochberg method. Based on the methylation profiles of cell‐type specific CpGs, blood cell composition was estimated (Jaffe & Irizarry, 2014). Correspondence analysis was performed as implemented in the vegan package.

At the individual CpG level, methylation differences between patients and controls were modeled using a linear model in the limma framework (Ritchie et al., 2015), adjusting for age, gender, and cell composition. The scores of the first two axes of a correspondence analysis on the estimated cell composition were included in the linear model to account for differences in cell composition. In a second step, we additionally introduced and tested an interaction term between methylation and age. Here, we investigated whether we could detect a different linear association (i.e., different slope) of age and methylation percentage between the classical WS and control groups. In contrast to both covariates (WS/CTRL or age) alone, the effect of the interaction term between aging and WS showed only a very weak signal on a genome‐wide scale. After multiple testing corrections, there were no significant sites left.

To derive DMRs from probewise *p* values, we used the approach implemented in the comb‐p package (Pedersen, Schwartz, Yang, & Kechris, 2012). First, a Stouffer–Liptak–Kechris (SLK)‐corrected *p* value for each probe was calculated based on the autocorrelation on neighboring *p* values. In a second step, regions enriched with SLK‐corrected *p* values were identified by a peak‐finding algorithm. Finally, the significance of each identified region was then determined by applying a Stouffer–Liptak correction to the original *p* values of all probes in the region. To correct for multiple testing, a Sidak correction, based on the number of possible regions of the same size, was applied to all identified regions. A region was extended if another *p* value within a genomic distance of 1,000 nucleotides was found (dist = 1,000). Sites with *p* < 0.05 (seed = 0.05) were considered as a starting point for a potential region.

Functional relevance of the genes covered by DMRs was analyzed using the Database for Annotation, Visualization and Integrated Discovery (DAVID), version 6.8 (https://david.ncifcrf.gov/). *p* Values for enrichment were calculated using Fisher's exact test and corrected for multiple testing with the Benjamini–Hochberg procedure.

### 4.4 Differential methylation and expression

Our 613 DMR genes associated with classical WS were compared with gene lists from five transcriptome studies of WS or *WRN*‐depleted cells (Table S3). The web tool GEO2R (https://www.ncbi.nlm.nih.gov/geo/geo2r/) with default options was used to compare the expression profiles (NCBI GEO GSE48761) between three WS fibroblast strains (GSM1184266‐GSM1184269, GSM1184272, and GSM1184273) and three controls (GSM1184256, GSM1184257, and GSM1184260–GSM1184263) from one of these studies (Cheung et al., 2014). Filtering for fold change >1.5 (logFC > 0.585) identified 970 differentially expressed genes.

## CONFLICT OF INTEREST

None declared.

## AUTHOR CONTRIBUTIONS

A.M., J.F., and I.N. performed experiments. M.D. and T.M. performed bioinformatic analyses. J.O. and G.M.M. recruited patients and provided study samples. A.M., M.P., and T.H wrote the manuscript. J.O., G.M.M., M.P., and T.H. critically reviewed and edited the manuscript.
