# Supplementary material for: Epigenetic signatures of Werner syndrome occur early in life and are distinct from normal epigenetic aging processes
Source: Aging Cell. 2019 Jul 1;18(5):e12995. doi: 10.1111/acel.12995 (PMC6718529; doi:10.1111/acel.12995)
Supplement: Supplementary file 1 [file ACEL-18-e12995-s001.docx]

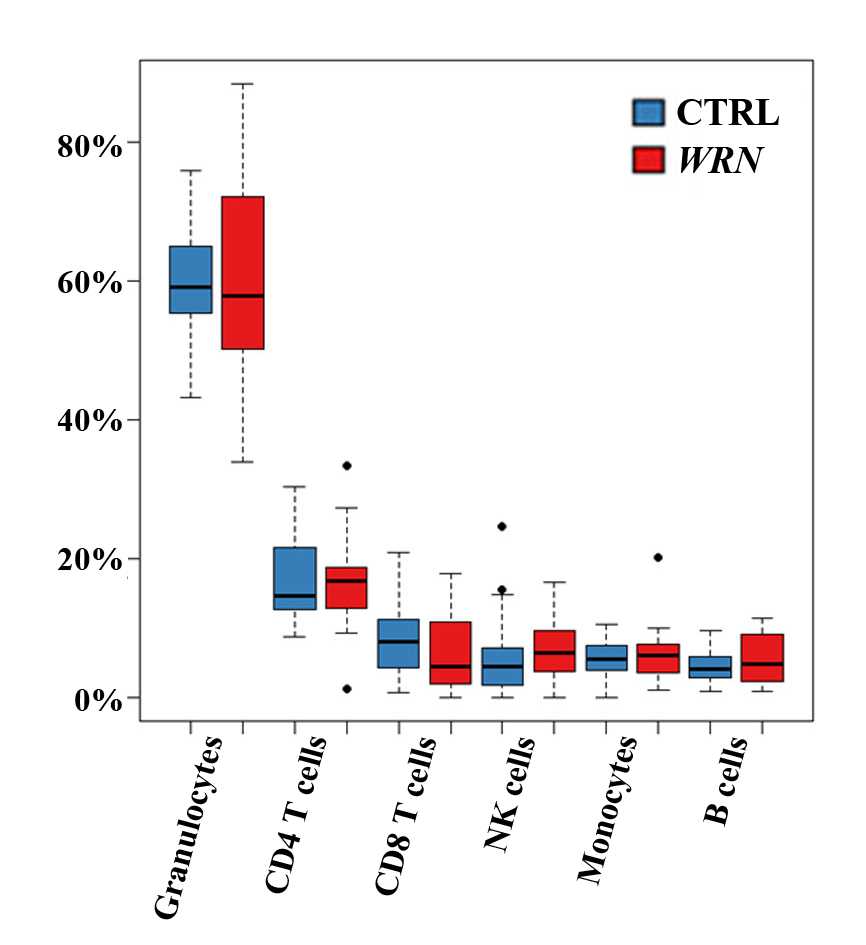


**Supporting information Figure S1**. Box plots of estimated blood-cell compositions for classical WS patients and controls. No significant (two-sample Wilcoxon test; p = 0.77 for granulocytes, p = 0.78 for monocytes, p = 0.68 for CD4 T, p = 0.15 for CD8 T, p = 0.46 for NK, and p = 0.62 for B cells) between-group differences were identified.

**
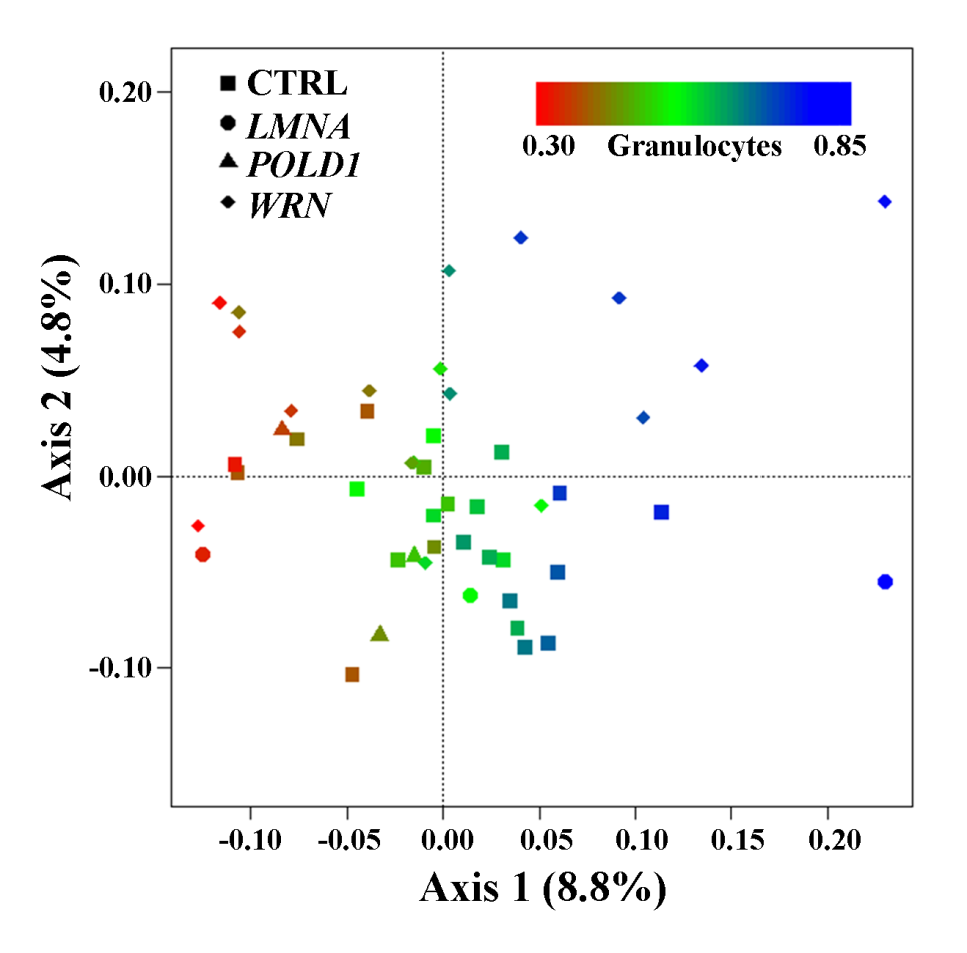
**

**Supporting information Figure S2.** Correspondence analysis of the 10,000 most variable CpG sites over all 48 blood samples. There is a clear granulocyte effect along the first axis, explaining 8.8% of the variance. The percentage of granulocytes is indicated by a color gradient from red to blue.

**Supporting information Table S1.**

Analysis of repeat methylation using a multivariable linear model adjusting for patient age.

| **Classical WS patients versus controls** | | | | |
| --- | --- | --- | --- | --- |
| **Amplicon** | **Estimate** | **Std.error** | **Statistic** | **P value** |
| Alpha-satellite DNA | -0.018 | 0.369 | -0.050 | 0.961 |
| ALU | 0.296 | 0.286 | 1.034 | 0.307 |
| LINE1 | 0.249 | 0.349 | 0.715 | 0.479 |
| rDNA region 1 | 1.410 | 1.511 | 0.933 | 0.356 |
| rDNA region 2 | 1.119 | 1.868 | 0.599 | 0.552 |
|  | | | | |
| **Global effect over the different WS genes** | | | | |
| **Amplicon** | **Sum Sq.** | **Df.** | **F value** | **P value** |
| Alpha-satellite DNA | 0.320 | 3 | 0.063 | 0.979 |
| ALU | 1.253 | 3 | 0.408 | 0.748 |
| LINE1 | 3.408 | 3 | 0.792 | 0.505 |
| rDNA region 1 | 34.886 | 3 | 0.446 | 0.721 |
| rDNA region 2 | 45.442 | 3 | 0.351 | 0.788 |

**Supporting information Table S3.** Gene expression studies in WS-patient derived and WRN-depleted cells vs. controls.

| **Reference** | **Analyzed cell type** | **Method** | **Gene lists in the reference** | **Number of differen-tially expressed genes** |
| --- | --- | --- | --- | --- |
| Kyng et al., 2003 | WS fibroblasts | Microarray | Suppl. Table 6A and 6C | 240 |
| Turaga et al., 2009 | WRN-depleted fibroblasts | Microarray | Suppl. Table 1 | 660 |
| Cheung et al., 2014 | WS fibroblasts | Microarray | GEO2R (GSE48761) | 970 |
| Zhang et al., 2015 | WRN-deficient mesenchymal stem cells | RNA Seq | Table S2 | 1,047 |
| Tang et al., 2016 | WS fibroblasts | Microarray | Tables S2 and S5 | 1,259 |
|  | WRN-depleted fibroblasts | Microarray | Tables S3 and S5 | 1,605 |

Cheung, H.H., Liu, X., Canterel-Thouennon, L., Li, L., Edmonson, C., & Rennert, O.M. (2014). Telomerase protects Werner syndrome lineage-specific stem cells from premature aging. *Stem Cell Reports*, 2, 534-546.

Kyng, K.J., May, A., Kolvraa, S., & Bohr, V.A. (2003). Gene expression profiling in Werner syndrome closely resembles that of normal aging. Proceedings of the National Academy of Sciences *of the United States of America*, 100, 12259-12264.

Tang, W., Robles, A.I., Beyer, R.P., Gray, L.T., Nguyen, G.H., Oshima, J., … Monnat Jr., RJ. (2016). The Werner syndrome RECQ helicase targets G4 DNA in human cells to modulate transcription. *Human Molecular Genetics*, 25, 2060-2069.

Turaga, R.V., Paquet, E.R., Sild, M., Vignard, J., Garand, C., Johnson, F.B., … Lebel, M. (2009). The Werner syndrome protein affects the expression of genes involved in adipogenesis and inflammation in addition to cell cycle and DNA damage responses. *Cell Cycle*, 8, 2080-2092.

Zhang, W., Li, J., Suzuki, K., Qu, J., Wang, P., Zhou, J., … Belmonte, J.C. (2015). Aging stem cells. A Werner syndrome stem cell model unveils heterochromatin alterations as a driver of human aging. *Science*, 348, 1160-1163.

**Supplementary Table S4.** Study samples.

| **Patient ID** | **Age** | **Gender** | **Country (Ethnicity)** | **Mutation 1** | **Mutation 2** |
| --- | --- | --- | --- | --- | --- |
|  |  |  |  |  |  |
| *WRN*-mutant 1 | 18 | male | German (Caucasian) | c.1105C>T | c.3961C>T |
| *WRN*-mutant 2 | 22 | male | India | c.561A>G | c.561A>G |
| *WRN*-mutant 3 | 32 | male | Dutch | c.1105C>T | c.1586G>A |
| *WRN*-mutant 4 | 37 | male | Canadian (Caucasian) | c.724+1G>T | c.2055delG |
| *WRN*-mutant 5 | 37 | male | India | c.2855C>A | c.2855C>A |
| *WRN*-mutant 6 | 37 | male | South Africa (Caucasian) | c.3590delA | c.3590delA |
| *WRN*-mutant 7 | 38 | male | New Zealand | c.1165delA | c.2089-1G>C |
| *WRN*-mutant 8 | 39 | male | Puerto Rican | c.1105C>T | c.1105C>T |
| *WRN*-mutant 9 | 40 | male | USA (Caucasian) | c.1105C>T | c.1105C>T |
| *WRN*-mutant 10 | 43 | male | Puerto Rican | c.1105C>T | c.1105C>T |
| *WRN*-mutant 11 | 43 | male | Japanese | c.1105C>T | c.3139-1G>C |
| *WRN*-mutant 12 | 45 | male | Afgani | c.2967+1G>A | c.2967+1G>A |
| *WRN*-mutant 13 | 45 | male | Poland | c.1105C>T | Unknown |
| *WRN*-mutant 14 | 47 | male | USA (Caucasian) | c.1578delC | c.2221C>T |
| *WRN*-mutant 15 | 49 | male | Turk | c.3234-160A>G | c.3234-160A>G |
| *WRN*-mutant 16 | 31 | female | USA (Hispanic) | c.3913C>T | c.3913C>T |
| *WRN*-mutant 17 | 36 | female | Turk | c.724G>C | c.724G>C |
| *WRN*-mutant 18 | 59 | female | Turk | c.3493C>T | c.3493C>T |
|  |  |  |  |  |  |
| *LMNA*-mutant 1 | 36 | male | USA (Caucasian) | c.1968G>A |  |
| *LMNA*-mutant 2 | 30 | female | USA (Caucasian) | c.1968G>A |  |
| *LMNA*-mutant 3 | 37 | female | Greek | c.1398T>A |  |
|  |  |  |  |  |  |
| *POLD1*-mutant 1 | 9 | female | German | c.1812_1814delCTC |  |
| *POLD1*-mutant 2 | 13 | female | Columbia | c.1812_1814delCTC |  |
| *POLD1*-mutant 3 | 30 | female | USA (Caucasian) | c.1812_1814delCTC |  |
